# Climate Effects and Feedback Structure Determining Weed Population Dynamics in a Long-Term Experiment

**DOI:** 10.1371/journal.pone.0030569

**Published:** 2012-01-17

**Authors:** Mauricio Lima, Luis Navarrete, José Luis González-Andujar

**Affiliations:** 1 Center for Advanced Studies in Ecology and Biodiversity (CASEB), Pontificia Universidad Católica de Chile, Santiago, Chile; 2 Departamento de Investigación Agraria, Instituto Madrileño de Investigación y Desarrollo Rural, Agrario y Alimentario (IMIDRA), Alcalá de Henares, Spain; 3 Departamento Protección de Cultivos, Instituto de Agricultura Sostenible, Consejo Superior de Investigaciones Científicas (CSIC), Córdoba, Spain; 4 Laboratorio Internacional de Cambio Global, LINCG (CSIC-PUC), Santiago, Chile; Ohio State University, United States of America

## Abstract

Pest control is one of the areas in which population dynamic theory has been successfully applied to solve practical problems. However, the links between population dynamic theory and model construction have been less emphasized in the management and control of weed populations. Most management models of weed population dynamics have emphasized the role of the endogenous process, but the role of exogenous variables such as climate have been ignored in the study of weed populations and their management. Here, we use long-term data (22 years) on two annual weed species from a locality in Central Spain to determine the importance of endogenous and exogenous processes (local and large-scale climate factors). Our modeling study determined two different feedback structures and climate effects in the two weed species analyzed. While *Descurainia* s*ophia* exhibited a second-order feedback and low climate influence, *Veronica hederifolia* was characterized by a first-order feedback structure and important effects from temperature and rainfall. Our results strongly suggest the importance of theoretical population dynamics in understanding plant population systems. Moreover, the use of this approach, discerning between the effect of exogenous and endogenous factors, can be fundamental to applying weed management practices in agricultural systems and to controlling invasive weedy species. This is a radical change from most approaches currently used to guide weed and invasive weedy species managements.

## Introduction

Population dynamics theory has been maturing during the last decades and nowadays we can explain the apparently complex numerical fluctuations exhibited by natural populations by means of a few general principles or laws [1]–[4]. One of the most important consequences of the existence of laws in population ecology is that models used to explain and predict ecological populations are based on these general principles [4], [5]. Indeed, population dynamics models constructed under that theory can be very useful for solving applied issues because of the fundamental role of models for predicting and explaining ecological systems [6]. Given the increasing need for conservation of endangered species, the management of exploited populations or the control of pests and invasive species, population ecology will have a great deal of social and political significance in facing the future challenges of global change. Therefore, it is likely that societal demands for practical applications of ecological theory will increase in the near future. To be successful, such applications will need to be based upon models that have proven their worth through empirical verification of their predictions [7].

Understanding the population dynamics of plants is fundamental to our ability to manage and predict ecosystem response, especially in the light of human alteration of climate. Although pest control is one of the areas in which population dynamics theory has been applied to solve practical problems [6], the links between population dynamics theory and model construction have been less emphasized in the management and control of weed populations and invasive weedy species [8].

Most management models on weed populations dynamics have emphasized the role of endogenous process, i.e., those capable of causing changes in a dynamical variable and are also affected in return by these changes, such as intra-specific competition, as being the most important factors driving the population dynamics [9]–[11]. These models produce stable dynamics and form the basis for weed management recommendations, yet exclude the role of the exogenous variables, i.e., those influencing the response of a determined variable but without being affected back by those changes, such as climate. To our knowledge, there are no other studies attempting to understand how both feedback structure and exogenous factors interact in shaping the dynamics of weed populations and their management.

Here, we use one of the longest data set (22 years) in plant populations on two annual weed species from a locality in Central Spain to determine the importance of endogenous (inter-specific interactions) and exogenous processes (climate). We focus on diagnosis and modeling tools from population-dynamics theory to analyze these long-term data and to determine the role of the North Atlantic Oscillation (NAO) and local weather as exogenous factors influencing weed dynamics. In particular, we use the Royama [1] classification of exogenous effects as an organized approach to evaluating the effect of climate on population dynamics. In this way, we can include logical explanations of the possible effects of climate on demographic rates in the population dynamics models and also use independent data for testing model predictions.

## Materials and Methods

### Site and sampling

A 22 year study (1985–2007) was conducted at El Encin Experimental Station (Alcala de Henares), Madrid, Spain (latitude: 40° 29′ N, Longitude: 3° 22′ W; Altitude: 610 m) using a cropping system based on a 2-year cereal-legume rotation. The experimental site has a north-Mediterranean climate, with mild and humid winters and dry-hot summers Average annual rainfall during the 22-year study period was 480 mm, with a maximum precipitation of 670 mm and a minimum of 230 mm. The average temperature was 13.1°C. All crops were grown under no-tillage and minimum tillage practices, maintaining plant residues close to the soil surface. During the wheat rotation phase, fertilizers were applied at relatively high rates (76 kg N, 120 kg P, 40 kg K ha ^−1^). Herbicides were sprayed for control of dicotyledonous weeds. No fertilizers or herbicides were applied in the legume rotation phase. Detailed information about the experiment is given in [12].

Weed population densities were sampled annually except in 1990 and 1997. The sampling times and procedures used to quantify weed population density varied slightly depending on the type of crop (cereal or legume) and the weed population density. In the wheat rotation phase, sampling took place from February to March. In the legume rotation phase sampling was slightly later (March to April). In the first three years of the experiment 5 destructive samples (30×33 cm) were taken along an M shape itinerary in each plot (20×40 m in size). Thereafter, 10 samples were taken in each plot except in 1995 when 20 samples were taken (10 along each of two transects). The collected material was kept in plastic bags and transported to the laboratory, where individual species were identified and counted. The different sampling intensities among years were due to the different weed densities present.

In this paper we consider two important species in cereal agro-ecosystems: *Descurainia sophia L*. (flixweed) and *Veronica hederifolia L.* (ivy-leaf speedwell). Both species have winter annual life histories with persistent seed banks and are relatively common in winter cereal crops grown in semi-arid areas [13], [14].

#### Diagnosis and statistical models of population dynamics

Population dynamics of weeds are the result of the combined effects of feedback structure (ecological interactions within and between plant populations), limiting factors (nutrient and water limitation), climatic influences (rainfall, temperature) and stochastic forces [15]. To understand how these factors may determine weed fluctuations, we model both system-intrinsic processes and exogenous influences as a general model based on the *R*-function [2]. The *R*-function represents the realized per capita population growth rates that synthesize the processes of individual survival and reproduction [2]. Defining *R_t_ = log (N_t_) – log (N_t-1_)*, we can express the *R*-function as
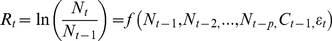
(1)where *N_t_* represents the weed abundance at time *t.* This model represents the basic feedback structure and integrates the stochastic and climate forces that drive population dynamics in nature. Our first step was to estimate the order of the dynamical processes in eqn. 1, that is, how many time lags, *N_t-i_*, should be included in the model for representing the feedback structure. First-order negative feedback processes are the results of intra-population interactions which involves a single variable (the density of population itself) due to the intra-specific competition for limiting resources [1]–[3]. Second-order feedback processes are produced by mutual causal process between two populations (consumer-resource; predator-prey; host-parasitoid), because two variables are now involved in the negative loop, it is known as a second-order dynamic process [1]–[3] and it had been demonstrated that this system can be reduced to a second-order or lagged equation for one of the two species involved [1]–[4]. To estimate the order of the process we used the partial rate correlation function, *PRCF*(*i*) [2], between *R_t_* and ln(*N_t_*_-*i*_) = *X_t-i_* after the effects of shorter lags have been removed. We write eqn. 1 in logarithmic form to calculate the partial correlations.
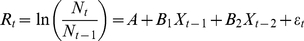
(2)


Where *R*, the realized per-capita rate of change, is calculated from the data,. We used a script written in the program R (R Development Core Team 2007) to calculate *PRCF _t-d_*. For statistical convenience we assumed a log-linear relationship between *R* and lagged population density [1]. Moreover, to perform the time series analysis, data were detrended by adjusting a linear model of the form *X_t_ = b + ft*, where *b* and *f* are the estimated parameters of the model. We used the residuals of this model plus the mean logarithm of density as the detrended time series. In order to test model predictions we make the time series of no-tillage and minimum tillage treatments comparable by subtracting or adding the differences between the means of the detrended time series.

#### Theoretical models of weed population dynamics

Population dynamics of weeds have been suggested to be the result of intra-population processes which cause a first-order feedback structure in plant populations [15]. To understand how these processes determine weed dynamics, we used a simple model of intra-specific competition, the exponential form of the discrete logistic model [2], [16], and we employed its generalized version;
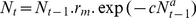
(3)


In this model *r_m_* is a positive constant representing the maximum finite reproduction rate, *c* is a constant representing competition and resource depletion, and *a* indicates the effect of interference on each individual as density increases [2]; *a*>1 indicates that interference intensifies with density and *a*<1 indicates habituation to interference. We can defining the above equation in terms of the *R*-function, i.e. 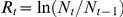, by log transforming equation 3 and defining the population density in logarithm *X_t_* = ln(*N_t_*), we obtain;
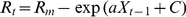
(4)where *R_m_* = ln(*r_m_*), *a* is the same parameter as in equation 3, *C* = ln(*c*), and *X* = ln(*N*). This model represents the basic feedback structure determined by intra-population processes.

Because in this model the three parameters *R_m_*, *a* and *C* have an explicit biological interpretation we can include climate perturbations in each parameter using the framework of Royama [1]. In this manner, we can build mechanistic hypotheses about the effects of climate on weed populations.

For example, simple additive rainfall perturbation effects can be represented as “vertical” effects, which shift the relative position of the *R*-function by changing *R_m_* on the *y* axis [1]. This can be expressed as:
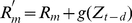
(5)


Where *g* is a simple linear function (+ or -) of some climate factor (Z) with different lags. Another kind of climate perturbation is when the equilibrium point of the population is influenced by the climate. This is the case when climate influences a limiting factor or resource (water, light or nutrients). The correct model structure in this scenario is that the carrying capacity (equilibrium point) is affected by the rainfall. In this case, the climate factor shifts the *R*-function curve along the *x*-axis without changing the slope at the equilibrium, which represents a “lateral” perturbation in the Royama [1] framework;
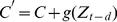
(6)


A previous study determined that the species *D. sophia* showed second-order oscillations [17], therefore our starting model was a second-order logistic model instead of the model from equation 3. A second-order logistic model can be represented as:
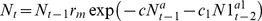
(7)


As in the equation 3, *N_t-d_* represents the lagged weed densities, *r_m_* is a positive constant representing the maximum finite reproduction rate, *c* is a constant representing competition and resource depletion, *a* indicates the effect of interference on each individual as density increase [1]. Similar to eqn. 4, we defined eqn. 8 in terms of the R-function resulting in the following equation:
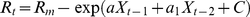
(8)where *a* and *a_1_* are the same parameters as in equation 7, and *C* = ln(*c + c_1_*).

We fitted equations 4 and 8 using the *nls* library in the program R by means of nonlinear regression analyses [18]. In addition, we included the climate variables in the parameters *R_m_*, *C* and *a* as linear functions (eqs. 5 and 6). All the models were fitted by minimizing the AIC_c_ = −2log(likelihood) + 2*p* + 2*p*(*p*+1)/(*n*-*p*-*1*), where *p* is the number of model parameters and *n* is the sample size. Models with lowest AIC_c_ values were selected. We fitted models to the time series of no-tillage data and tested model predictions in the minimum tillage data time series, in addition we repeated this procedure in the opposite manner, fitting models in minimum tillage time series data and comparing the predictions in the no-tillage time series data. Observed and predicted dynamics was compared using a bias parameter, calculated as ∑(*O_i_* – *P_i_*)/n where *O_i_* is observed data and *P_i_* is predicted data and the Pearson's correlation coefficient between observed and predicted data. Because the models of *Veronica hederifolia* showed no convergence, we use biological criteria for fixing the *Rm* parameter (maximum per capita growth rates) [1]. The maximum value observed of the per capita growth rate was 2.5; we fixed this value in 3 for estimating the other model parameters.

## Results

**Figure 1:**
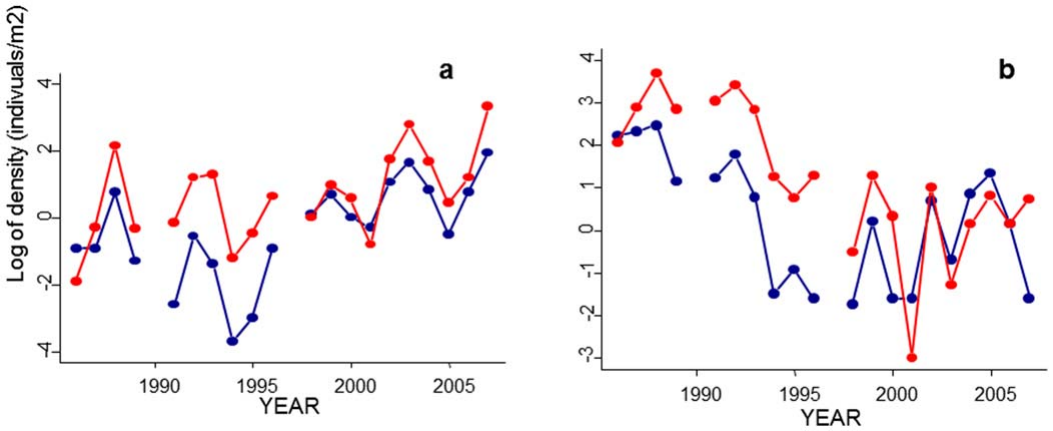
Observed numerical fluctuations (ln of number of individuals/m^2^) of the two weed species; a) *Descurainia Sophia*; and b) *Veronica hederifolia* for the no-tillage (blue dots and line) and minimum tillage (red dots and line) systems.

**Figure 2:**
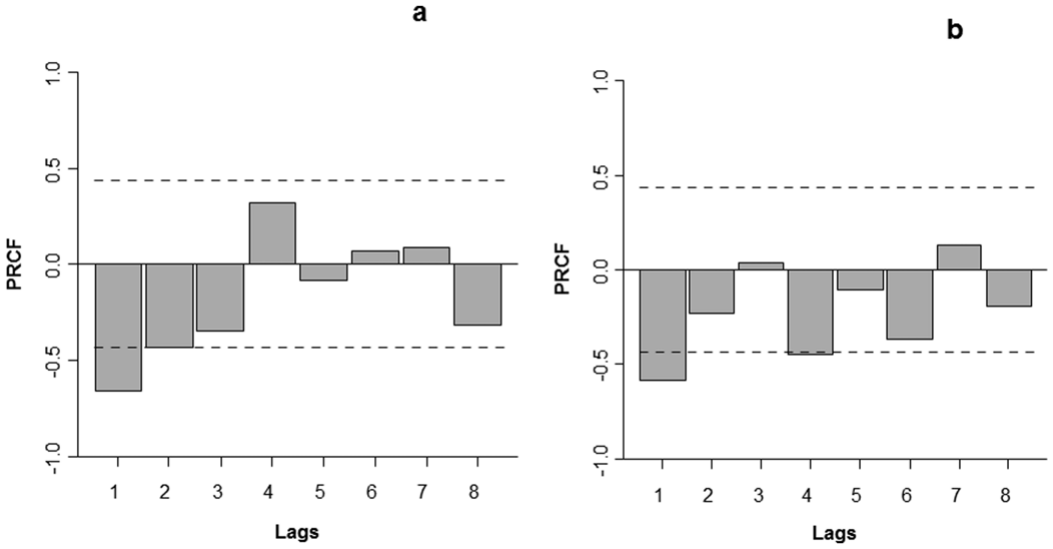
Partial rate correlation functions for transformed data. The major influence of first-order feedback structure is clear in the species *Veronica hederifolia (b)* while a second-order structure is diagnosed for the species *Descurainia Sophia (a).* The dotted line in the figure show the interval of ± 2 SD calculated with Bartletts formula.

The numerical fluctuation of *D. sophia* was characterized by regular periodic oscillations and a positive trend (Figure 1a). *V. hederifolia* was characterized by irregular oscillations and a clear negative trend (Figure 1b). The differences between species in the dynamic pattern were associated with the relative importance of first- and second-order feedbacks: while the per capita growth rate of *D. sophia* showed a second-order effect, *V. hederifolia* was characterized by a first-order feedback (Figure 2a and b).

Results from the model fitting showed that the maximum per capita population growth rates, *Rm*, varied between 3 and 4 in the two species indicating the high potential for population growth of both weeds (Table S1). In *V. hederifolia* the pure endogenous model explained 30% and 49% of the variability in per capita growth rates in the no-tillage and minimum tillage systems (Table S1). Exogenous effects improved the explained variance of the pure endogenous models by an average of 22% in no-tillage, ranging from 1 to 46%, and 12% in minimum tillage, ranging from 0 to 20%. Model 7, which included rainfall and summer temperature, performed best among our candidate models in no-tillage system with a AIC_c_ of 48.91 (Table S1). Rainfall and summer temperature positively influenced population growth rate of *V. hederifolia.* In the minimum tillage system, model 9 including the NAO effects was the best with AIC_c_ = 57.65 (Table S1). NAO negatively influenced the population growth rate (Table S1).

**Figure 3:**
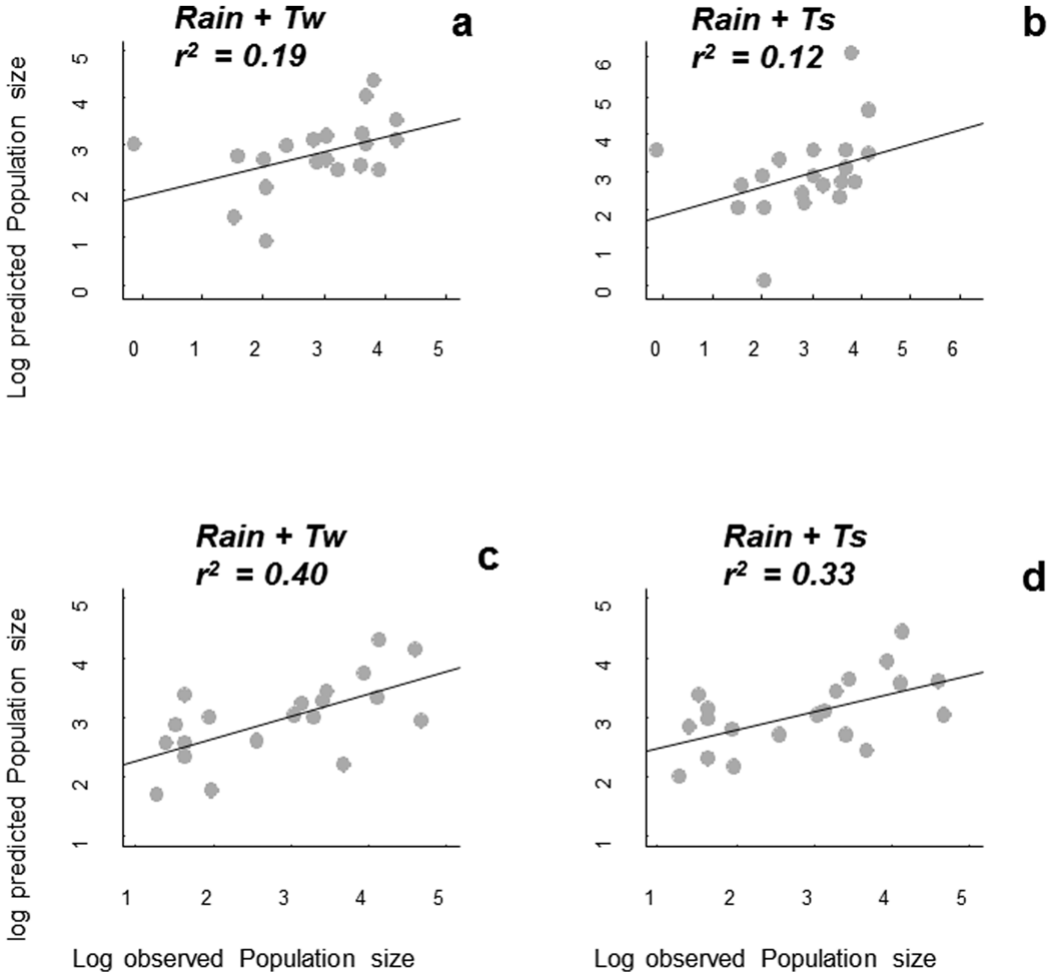
Upper row: comparison of observed *Veronica hederifolia* population densities in the minimum-tillage system versus predicted densities from models fitted to the data from the no-tillage system; lower row: comparison of observed *Veronica hederifolia* densities in the no-tillage system versus predicted densities to the data from the minimum tillage system. a) Model 6, b) model 7, c) model 13, d) model 15. All models are from Table S1.

**Figure 4:**
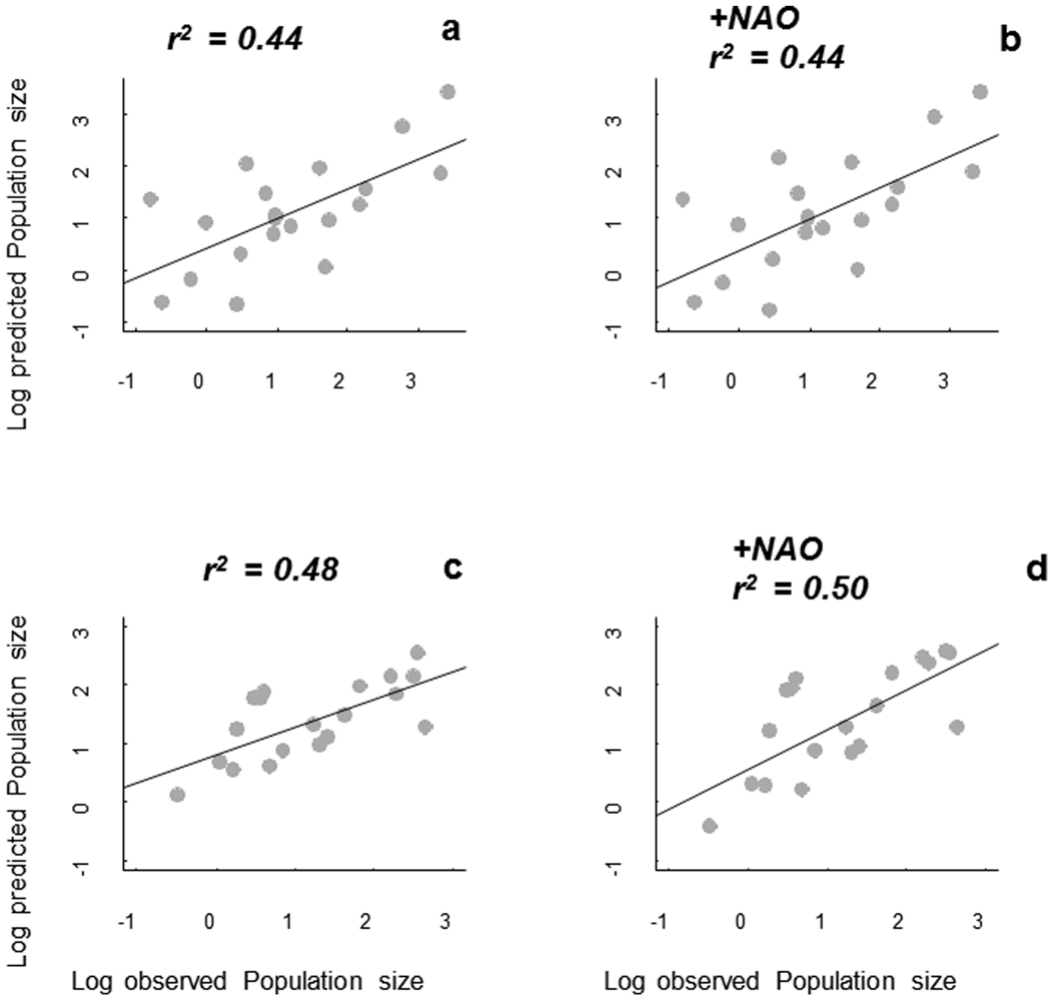
Superior row; comparison of observed *Descurainia sophia*'s densities in the minimum-tillage system versus predicted densities from models fitted to the data from the no-tillage system; inferior row; comparison of observed *Descurainia sophia*'s densities in the no-tillage system versus predictions from models fitted to the data from the minimum tillage system. a) Model 17, b) model 18, c) model 22 and d) model 23. All models are from Table S1.

From a predictive point of view, *V. hederifolia* models that include rainfall and winter temperature were better than models including rainfall and summer temperature (Fig. 3). In addition, models fitted to minimum-tillage data sets were better predictors of observed data in no-tillage systems than *vice versa* (Fig. 3; Table S1). The second-order logistic model for *D. sophia* explained 80% and 65% of the observed variation in per capita growth rates in the no-tillage and minimum tillage systems, respectively (Table S1). Exogenous effects improved the explained variance of the pure endogenous models by an average of 1% in no-tillage, ranging from 0 to 2%, and 5.5% in minimum tillage, ranging from 0 to 14%. In the no-tillage data set, the pure endogenous second-order model showed the best fit with AIC_c_ = 37.90 (Table S1). In the minimum-tillage data set, the model including the NAO effects was the best one with AIC_c_ = 47.88 (Table S1). However, in both data sets the model predictions using the pure endogenous second-order models were very similar to those using an exogenous model including NAO (Fig. 4; Table S1).

## Discussion

Our modeling study determined two different feedback structures in the two weed species analyzed. While *D.* s*ophia* exhibited a second-order feedback and low climate influence, *V. hederifolia* was characterized by a first-order feedback structure and important effects of climate variables. The endogenous structure therefore appears to be stronger in *D.* s*ophia* than in *V. hederifolia.*

The dynamics of *D.* s*ophia* were mainly explained by endogenous factors. A second order feedback structure – delayed density dependence – captured the essential elements of the population dynamics of this species in both minimum and no-tillage (Table S1). It has been suggested that the accumulation of plant litter as a consequence of high nutrient levels might be a plausible explanation for the second-order feedback structure found in *D. sophia* under no-tillage practice [17]. Growth of *D. sophia* in that study took place in a cropping system with high nutrient levels. High nutrient supply could lead to high crop and weed biomass production and high rates of crop litter deposition [19]. The accumulation of plant litter in the topsoil resulting from no-tillage and reduced tillage systems may potentially cause important changes to the physical and chemical environment of the soil surface and may act as a time-delayed inhibitor on the germination of *D. sophia* populations [20], [21].

Exogenous factors contributed little to the dynamics of *D. sophia.* Local climate factors did not have any significant influence on *D. sophia* population dynamics, whereas NAO was more determinant. In the minimum tillage system, models including NAO and delayed density dependence (model 23, Table S1) produced the more plausible model and explained the higher variability (79%). However, the importance of NAO was different in no-tillage and minimum tillage systems (Table S1). NAO had a clearer influence in minimum tillage. It was probably due to the NAO negative effect (Table S1) increasing precipitations in the Mediterranean area. Minimum tillage produces a small soil disturbance, affecting soil microenvironments due to differences in soil porosity, bulk density and soil surface conditions. Thus, minimum tillage provides less moisture conservation than no tillage and plants under this tillage system would need additional moisture provided by climate factors, especially in a semi-arid climate. It is interesting to note that the predictive capacity of model 23 was similar whether or not it included NAO (Table S1). Our results indicated that *D. sophia* presented low sensitivity to local climate effects, such as precipitation and temperature. These results are surprising because local climate factors are considered to be determinant in weed emergence [22]. This is especially true in Mediterranean climates, where water availability is the most important environmental constraint, due to the combination of high summer temperatures and low rainfall [23].

In contrast to the environmental independence of models of *D. sophia* population dynamics, the pure endogenous model for *V. hederifolia* per capita growth rates explained less than 49% of the variability in both the no-tillage and minimum tillage systems (Table S1). Population dynamics of *V. hederifolia* seemed to be driven mainly by climate factors. Large-scale and local scale exogenous factors had a different role in the growth rates of this species. Under no tillage the main driving force was the local weather (rainfall and temperature) (Table S1). Regarding the minimum tillage system, NAO seemed to have the main role (Table S1). It was noticeable that the importance of NAO was higher in the minimum tillage system for both species. However, the best predictions are from the model including winter temperature and rainfall fitted to the minimum tillage system and used to predict no-tillage data (Table S1). In contrast, models fitted to the no-tillage system did not predict the data from minimum tillage system very well. One potential explanation for this pattern is that no-tillage system appears to be more influenced by exogenous variables (see Table S1). Therefore the parameter values from models fitted on data from this system can have more source of unknown variation.

Two different patterns emerge from our results. On the one hand, exogenous factors seem to mainly influence the population dynamics of *V. hederifolia,* in agreement with the general view in weed science [24]. On the other hand, endogenous factors seem to be the main driver of the population dynamics of *D. sophia*. The use of this approach, discerning between the role of exogenous and endogenous factors, can be fundamental to applying weed management practices in agricultural systems and controlling invasive weedy species. This approach signifies a radical change relative to most approaches currently used to guide weed management [8].

### Conclusions

The use of the population dynamics theory for modeling weed populations represents an important new approach to controlling weed populations, and therefore has a better chance of guiding suitable management recommendations. In this paper we used proper diagnosis analysis [2] and *a posteriori* modeling to deduce the potential causes of weed population fluctuations. Our results strongly suggest the importance of theoretical population dynamics to understand this system. Moreover, the use of this approach can be fundamental to applying weed management practices in agricultural systems. Understanding the interactions between endogenous and exogenous factors in shaping the dynamics of weed populations may have important implications for management of weed and invasive plants, climate change mitigation and biodiversity conservation in agro-ecosystems.

## Supporting Information

Table S1 *b* maximum finite reproductive rate, *a* non-linearity coefficient, *C* equilibrium point, *d, e* and *f* coefficients for different effects, *r^2^* coefficient of determination, *AIC_c_* Akaike information criterion corrected for small sample bias, *ΔAIC_c_* differences in *AIC_c_, likelihood exp(-ΔAIC_c_/2), k* number of estimated parameters**,** *R_t_ = ln(Nt)-ln(N_t-1_)* realized logarithmic per-capita population growth rate, *X_t-1_* logarithmic density, *NAO = North Atlantic Oscillation Index, TW* winter temperature, *P* precipitation. Models 1, 8, 17 and 22 represent endogenous effects only, the other models concider climate variables as exogenous effects. The most likely model (defined by the lowest *AIC_c_*) is highlighted in bold.
(DOC)
