# Supplementary material for: Climate Effects and Feedback Structure Determining Weed Population Dynamics in a Long-Term Experiment
Source: PLoS One. 2012 Jan 17;7(1):e30569. doi: 10.1371/journal.pone.0030569 (PMC3260292; doi:10.1371/journal.pone.0030569)
Supplement: Table S1 — b maximum finite reproductive rate, a non-linearity coefficient, C equilibrium point, d, e and f coefficients for different effects, r2 coefficient of determination, AICc Akaike information criterion corrected for small sample bias, ΔAICc differences in AICc, likelihood exp(-ΔAICc/2), k number of estimated parameters, Rt = ln(Nt)-ln(Nt-1) realized logarithmic per-capita population growth rate, Xt-1 logarithmic density, NAO = North Atlantic Oscillation Index, TW winter temperature, P precipitation. Models 1, 8, 17 and 22 represent endogenous effects only, the other models concider climate variables as exogenous effects. The most likely model (defined by the lowest AICc) is highlighted in bold. (DOC) [file pone.0030569.s001.doc]

Table 1. *b* maximum finite reproductive rate, *a* non-linearity coefficient, *C* equilibrium point, *d, e* and *f* coefficients for different effects, *r^2^* coefficient of determination, *AIC_c_* Akaike information criterion corrected for small sample bias, *ΔAIC_c_* differences in *AIC_c_, likelihood exp(-ΔAIC_c_/2), k* number of estimated parameters***,*** *R_t_=ln(Nt)-ln(N_t-1_)* realized logarithmic per-capita population growth rate, *X_t-1_* logarithmic density, *NAO = North Atlantic Oscillation Index, TW* winter temperature, *P* precipitation. Models 1, 8, 17 and 22 represent endogenous effects only, the other models concider climate variables as exogenous effects. The most likely model (defined by the lowest *AIC_c_*) is highlighted in bold.

| ***Models for weed dynamics*** |  |  |  |  |  |  |  |  |  |  |  |  |  |
| --- | --- | --- | --- | --- | --- | --- | --- | --- | --- | --- | --- | --- | --- |
| *Veronica sp* (Non-tillage) | ***b*** | ***a_1_*** | ***a_2_*** | ***C*** | ***D*** | ***e*** | ***R^2^*** | ***AIC_c_*** | ***∆AIC_c_*** | ***Loglik.*** | ***K*** | ***Bias*** | ***r (observed-predicted)*** |
| 1.$R_{t}=b-\exp(aX_{t-1}+C)$ | 3.0 | 0.21 |  | 0.51 |  |  | 0.30 | 59.37 | 10.46 | -25.76 | 3 |  |  |
| 2.$R_{t}=b-\exp(aX_{t-1}+C)+dNAO_{t}$ | 3.0 | 0.23 |  | 0.33 | -0.30 |  | 0.44 | 59.32 | 10.42 | -23.99 | 4 | 1.08 | 0.29 |
| 3.$R_{t}=b-\exp(aX_{t-1}+C)+dTW_{t}$ | 3.0 | 0.42 |  | -0.80 | -0.26 |  | 0.41 | 60.48 | 11.58 | -24.57 | 4 | 1.14 | 0.21 |
| 4.$R_{t}=b-\exp(aX_{t-1}+C)+dTS_{t}$ | 3.0 | 0.12 |  | 1.27 | 0.095 |  | 0.31 | 62.55 | 13.65 | -25.61 | 4 | 1.21 | -0.06 |
| 5.$R_{t}=b-\exp(aX_{t-1}+C)+dP_{t}$ | 3.0 | 0.08 |  | 1.46 | 0.008 |  | 0.59 | 53.78 | 4.88 | -21.22 | 4 | 1.10 | 0.34 |
| 6.$R_{t}=b-\exp(aX_{t-1}+C)+dP_{t}+eTW_{t}$ | 3.0 | 0.13 |  | 1.05 | 0.007 | -0.17 | 0.61 | 57.05 | 8.14 | -20.29 | 4 | 1.02 | 0.44 |
| 7.$R_{t}=b-\exp(aX_{t-1}+C)+dP_{t}+eTS_{t}$ | **3.0** | **0.004** |  | **2.99** | **0.013** | **0.62** | **0.76** | **48.91** | **0.00** | **-20.55** | **4** | 1.29 | 0.34 |
| *Veronica sp* (Minimum-tillage) | ***b*** | ***a_1_*** | ***a_2_*** | ***C*** | ***d*** | ***e*** | ***R^2^*** | ***AIC_c_*** | ***∆AIC_c_*** | ***Loglik.*** | ***K*** | ***Bias*** | ***r (observed-predicted)*** |
| 8.$R_{t}=b-\exp(aX_{t-1}+C)$ | 4.0 | 0.27 |  | 0.56 |  |  | 0.49 | 61.28 | 3.63 | -26.73 | 3 |  |  |
| 9.$R_{t}=b-\exp(aX_{t-1}+C)+d\cdot NAO_{t}$ | **4.0** | **0.26** |  | **0.47** | **-0.44** |  | **0.67** | **57.65** | **0.00** | **-23.16** | **4** | 1.22 | 0.23 |
| 10.$R_{t}=b-\exp(aX_{t-1}+C)+d\cdot TW_{t}$ | 4.0 | 0.40 |  | -0.24 | -0.24 |  | 0.56 | 62.88 | 5.23 | -25.77 | 4 | 1.07 | 0.36 |
| 11.$R_{t}=b-\exp(aX_{t-1}+C)+d\cdot TS_{t}$ | 4.0 | 0.23 |  | 0.89 | 0.038 |  | 0.49 | 64.67 | 7.02 | -26.67 | 4 | 1.22 | -0.31 |
| 12.$R_{t}=b-\exp(aX_{t-1}+C)+d\cdot P_{t}$ | 4.0 | 0.15 |  | 1.31 | 0.006 |  | 0.58 | 61.32 | 3.67 | -25.00 | 4 | 0.98 | 0.51 |
| 13.$R_{t}=b-\exp(aX_{t-1}+C)+d\cdot P_{t}+e\cdot TW_{t}$ | 4.0 | 0.22 |  | 0.75 | 0.005 | -0.25 | 0.63 | 63.98 | 6.33 | -24.26 | 5 | 0.88 | 0.64 |
| 14.$R_{t}=b-\exp(aX_{t-1}+C)+d\cdot NAO_{t}+e\cdot TW_{t}$ | 4.0 | 0.28 |  | 0.35 | -0.42 | -0.045 | 0.67 | 61.72 | 4.06 | -23.13 | 5 | 1.20 | 0.26 |
| 15.$R_{t}=b-\exp(aX_{t-1}+C)+d\cdot P_{t}+e\cdot TS_{t}$ | 4.0 | 0.10 |  | 1.82 | 0.007 | 0.11 | 0.59 | 66.26 | 8.61 | -25.40 | 5 | 0.93 | 0.58 |
| 16.$R_{t}=b-\exp(aX_{t-1}+C)+d\cdot NAO_{t}+e\cdot TS_{t}$ | 4.0 | 0.13 |  | 1.51 | -0.48 | 0.15 | 0.69 | 60.77 | 3.11 | -22.66 | 5 | 1.18 | 0.29 |
| *Descurainia sophia* (Non-tillage) | ***b*** | ***a_1_*** | ***a_2_*** | ***C*** | ***d*** | ***e*** | ***R^2^*** | ***AIC_c_*** | ***∆AIC_c_*** | ***Loglik.*** | ***K*** | ***Bias*** | ***r (observed-predicted)*** |
| 17.$R_{t}=b-\exp(a_{1}X_{t-1}+a_{2}X_{t-2}+C)$ | **3.0** | **0.33** | **0.28** | **0.34** |  |  | **0.80** | **37.90** | **0.00** | **-13.13** | **4** | 0.24 | 0.65 |
| 18.$R_{t}=b-\exp(a_{1}X_{t-1}+a_{2}X_{t-2}+C)+d\cdot NAO_{t}$ | 3.0 | 0.34 | 0.28 | 0.28 | -0.11 |  | 0.82 | 40.80 | 2.90 | -12.40 | 5 | 0.22 | 0.66 |
| 19.$R_{t}=b-\exp(a_{1}X_{t-1}+a_{2}X_{t-2}+C)+d\cdot TW_{t}$ | 3.0 | 0.33 | 0.27 | 0.37 | 0.0094 |  | 0.80 | 42.25 | 4.35 | -13.12 | 5 |  |  |
| 20.$R_{t}=b-\exp(a_{1}X_{t-1}+a_{2}X_{t-2}+C)\cdot d\cdot TS_{t}$ | 3.0 | 0.18 | 0.18 | 1.19 | 0.097 |  | 0.81 | 41.24 | 3.34 | -12.62 | 5 |  |  |
| 21.$R_{t}=b-\exp(a_{1}X_{t-1}+a_{2}X_{t-2}+C)+d\cdot P_{t}$ | 3.0 | 0.25 | 0.24 | 0.76 | 0.0022 |  | 0.81 | 41.00 | 3.10 | -12.50 | 5 |  |  |
| *Descurainia sophia* (Minimum-tillage) | ***b*** | ***a_1_*** | ***a_2_*** | ***C*** | ***d*** | ***e*** | ***R^2^*** | ***AIC_c_*** | ***∆AIC_c_*** | ***Loglik.*** | ***K*** | ***Bias*** | ***r (observed-predicted)*** |
| 22.$R_{t}=b-\exp(a_{1}X_{t-1}+a_{2}X_{t-2}+C)$ | 3.0 | 0.29 | 0.24 | 0.38 |  |  | 0.65 | 50.39 | 2.51 | -19.37 | 4 | 0.71 | 0.69 |
| 23.$R_{t}=b-\exp(a_{1}X_{t-1}+a_{2}X_{t-2}+C)+d\cdot NAO_{t}$ | **3.0** | **0.34** | **0.27** | **0.096** | **-0.35** |  | **0.79** | **47.88** | **0.00** | **-15.44** | **5** | 0.80 | 0.70 |
| 24.$R_{t}=b-\exp(a_{1}X_{t-1}+a_{2}X_{t-2}+C)+d\cdot TW_{t}$ | 3.0 | 0.40 | 0.38 | -0.37 | -0.16 |  | 0.68 | 53.69 | 5.81 | -18.85 | 5 |  |  |
| 25.$R_{t}=b-\exp(a_{1}X_{t-1}+a_{2}X_{t-2}+C)+d\cdot TS_{t}$ | 3.0 | 0.24 | 0.20 | 0.71 | 0.029 |  | 0.65 | 54.71 | 6.83 | -19.36 | 5 |  |  |
| 26.$R_{t}=b-\exp(a_{1}X_{t-1}+a_{2}X_{t-2}+C)+d\cdot P_{t}$ | 3.0 | 0.22 | 0.19 | 0.84 | 0.0036 |  | 0.70 | 52.82 | 4.94 | -18.41 | 5 |  |  |
